# How do you recognize and reconstitute a synaptic vesicle after fusion?

**DOI:** 10.12688/f1000research.12072.1

**Published:** 2017-09-22

**Authors:** Natali L. Chanaday, Ege T. Kavalali

**Affiliations:** 1 Department of Neuroscience, University of Texas Southwestern Medical Centre, Dallas, TX, 75390-9111, USA

**Keywords:** synaptic vesicle, transient fusion, kiss-and-run endocytosis, neurotransmission

## Abstract

Synaptic vesicle recycling is essential for sustained and reliable neurotransmission. A key component of synaptic vesicle recycling is the synaptic vesicle biogenesis process that is observed in synapses and that maintains the molecular identity of synaptic vesicles. However, the mechanisms by which synaptic vesicles are retrieved and reconstituted after fusion remain unclear. The complex molecular composition of synaptic vesicles renders their rapid biogenesis a daunting task. Therefore, in this context, kiss-and-run type transient fusion of synaptic vesicles with the plasma membrane without loss of their membrane composition and molecular identity remains a viable hypothesis that can account for the fidelity of the synaptic vesicle cycle. In this article, we discuss the biological implications of this problem as well as its possible molecular solutions.

## Introduction

In presynaptic nerve terminals, neurotransmitters are packed into small membranous organelles called synaptic vesicles. When the action potential arrives at the terminal, voltage-gated calcium channels open and the resulting rise in intrasynaptic Ca ^2+^ concentration leads to fusion of the synaptic vesicles with the plasma membrane, thus releasing their content (that is, the neurotransmitters). This fusion, also called exocytosis, occurs at a specialized area composed of a dense matrix of proteins termed the active zone. In addition, synaptic vesicles can fuse spontaneously in the absence of action potentials. Although the molecular fusion machinery involved in evoked and spontaneous release shows some differences ^1^, in both cases vesicles can be retrieved swiftly after fusion with the active zone membrane ^2– 4^.

Synaptic vesicles are complex organelles ^5^. They require multiple integral protein components to be functional (vesicular SNAREs, synaptotagmins, neurotransmitter transporters, V-ATPase, and so on). Surprisingly, the exact function of several characteristic synaptic vesicle proteins (such as the glycoprotein SV2) remains a mystery ^6, 7^. Synaptic vesicle proteins are rather heterogeneous in their molecular structure and contain no clear consensus targeting sequence, making it unclear whether they are recognized and recruited through a common pathway or, more likely, via diverse convergent mechanisms. Therefore, it is difficult to envision how vesicles can be reconstituted with specificity and rapidity unless synaptic vesicle proteins are nucleated by some form of protein-protein interactions ^8^, where certain vesicle components may form “hubs” to facilitate such nucleation ^9^. Many pathways of synaptic vesicle recycling have been proposed; here, we discuss the current knowledge about their molecular mechanisms and implications for fast neurotransmission, based on the premise of molecular identity preservation after fusion (for another complete review on presynaptic endocytosis mechanisms, see 10, 11).

## The problem of regaining molecular identity after full collapse fusion

**Figure 1.**
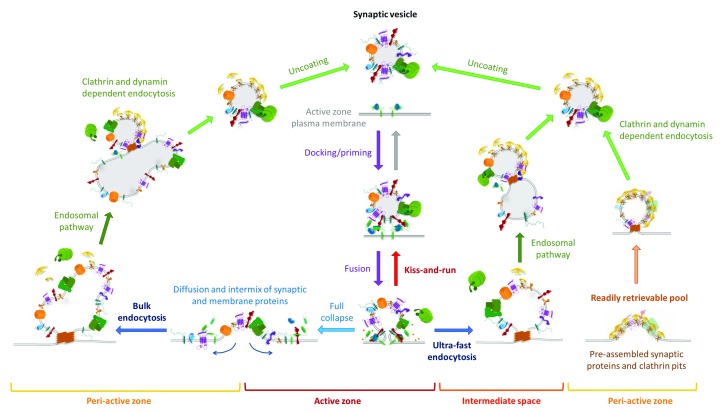
Summary of the synaptic vesicle cycle. After docking and priming (top center, purple arrows), synaptic vesicles are ready to fuse (either evoked or spontaneously) with the active zone membrane (bottom center, purple arrow). After fusion, vesicles can be rapidly retrieved without morphological or molecular changes by closure of the fusion pore (kiss-and-run, center, red arrow) or can fully collapse, irreversibly, intermixing its components with the plasma membrane (bottom left, light blue arrow). Synaptic vesicle proteins can be specifically retrieved from the plasma membrane via an ultra-fast mechanism (bottom right, blue arrow) or, during sustained stimulation, via a slower bulk endocytosis pathway (bottom left, blue arrow). Note that the endosomal-like intermediaries generated in both cases might not be the same (neither morphologically nor molecularly). Afterwards, synaptic vesicles are regenerated from the synaptic endosomes via a clathrin-dependent mechanism (left and right sides, green arrows). Finally, synaptic vesicle proteins can also be rapidly retrieved from pre-assembled clusters containing adaptor proteins and clathrin, which would be endocytosed upon stimulation in synchrony with neurotransmitter release (bottom right, orange arrow).

Classically, synaptic vesicles were thought to completely collapse onto the plasma membrane after fusion and subsequently vesicle membrane components (lipids as well as proteins) intermix with their plasma membrane counterparts. Afterwards, adaptor proteins—such as AP-2, stonin-2, and AP-180 ^11, 12^—bind to and cluster certain synaptic vesicle proteins, and also recruit clathrin and other partners, typically within the periphery of the active zone. The synaptic proteins synaptobrevin-2 and synaptophysin-1 were proposed to be required for the proper recruitment and trafficking of other synaptic vesicle proteins, and collectively they have been called intrinsic trafficking partners, or iTRAPs (for a complete review on this pathway, see 12). This clustering leads to the formation of coated vesicles which eventually bud off from the plasma membrane with the help of the GTPase dynamin ^13^. A V-type ATPase then lowers the pH in these vesicles (by pumping H ^+^ at the cost of ATP) and the resulting electrochemical gradient propels the refilling of synaptic vesicles with neurotransmitters. In this way, a whole synaptic vesicle is regenerated, with its characteristic membrane composition, completing the synaptic vesicle cycle. Some studies posed the possibility that the nervous system could have evolved a modified version of this classic clathrin-mediated endocytosis mechanism, more suitable for sustaining reliable neurotransmitter release. In this regard, a recent hypothesis proposes the existence of pre-assembled patches of synaptic vesicle components (lipids and proteins) at the periactive zone in a so-called “readily retrievable pool” which undergoes clathrin-mediated endocytosis upon membrane fusion ^14^ ( Figure 1). Regardless of the existence (or not) of pre-assembled protein clusters, the entire process for exocytosed vesicles to be re-available for release through a clathrin-mediated mechanism occurs within 4 to 90 seconds ^15^, significantly slower than the time course of neurotransmission, which is in the order of milliseconds. It is worth mentioning that, besides the plasma membrane, endocytosis of clathrin-coated vesicles may occur from membrane infoldings or endosomal cisternae, which form upon the accumulation of fused synaptic vesicles after strong and repetitive stimulation, a process called activity-dependent bulk endocytosis ^16^ ( Figure 1).

Recent innovative studies have proposed an ultra-fast mechanism of synaptic vesicle retrieval in small central synapses ^17^. According to this mechanism, following full collapse of synaptic vesicles with the active zone membrane (during the first roughly 30 ms after the stimulus), ultra-fast endocytosis occurs at the edges of the active zone, 50 to 100 ms after exocytosis, order(s) of magnitude (~200-fold) faster than the mean rate of direct clathrin-mediated endocytosis ^18^. In fact, the endocytosis step of this ultra-fast pathway is proposed to be clathrin-independent but mediated by actin and dynamin. Ultra-fast compensatory endocytosis is highly temperature-dependent, occurring only at near-physiological temperatures (~34°C), and increases proportionally with the number of fused vesicles. On a subsequent step (~1 second later), endosome-like structures are generated, from which small vesicles regenerate through a clathrin-dependent mechanism within 3 to 5 seconds ^19^ ( Figure 1). These findings explain, in a quantitative fashion, how synapses can undergo endocytosis at the same timescale of exocytosis, keeping the total presynaptic membrane surface constant, thus answering a long-standing dilemma in neurobiology. Nevertheless, the reconstitution of a functional, release-ready synaptic vesicle still requires several seconds, and so far there are no indications that the molecular identity of that vesicle will be the same as the one that originally fused. More likely, owing to full collapse fusion and mixing of membranous components, identity would not be preserved, leaving open the question about conservation of synaptic vesicle identity. Moreover, the readily retrievable pool hypothesis and the ultra-fast endocytosis mechanism both require the presence of a still-unknown intermediary to couple exocytosis and endocytosis given that they occur in spatially separate areas of the presynaptic membrane. The most fitting candidate for this role is the Ca ^2+^ concentration rise during activity. However, whereas Ca ^2+^ regulation of the fusion process has been widely described, its exact role in synaptic vesicle endocytosis remains poorly understood ^20^. At present, it is still a matter of debate whether Ca ^2+^ triggers endocytosis, whether it is absolutely necessary and sufficient for this task, or whether it only regulates kinetic or synchronicity aspects (or both) of synaptic vesicle retrieval.

Recent molecular and functional evidence suggests that a fast mode of clathrin-independent endocytosis in central synapses involves the formation of endosome-like intermediaries upon stimulation requiring dynamin, formin (an actin nucleation factor), myosin II, and actin function ^21^. This study also indicated that a wide range of endocytic timescales coexist at physiological temperature, suggesting that ultra-fast endocytosis might be saturated upon strong, repetitive stimulation and other endocytic pathways might take its place. In accordance with these findings, the retrieval of granules in secretory chromaffin cells is also clathrin-independent and is regulated by dynamin and F-actin polymerization ^22, 23^. Interestingly, the mode of dense-core vesicle exo-endocytosis is regulated by the amount of Ca ^2+^ influx, modulating the proportion of full fusion events or the rate of fusion pore closure ^22, 24^, suggesting that Ca ^2+^ may act as a regulator of different modes of exo- and endocytosis.

Regardless of whether synaptic membrane components are retrieved directly via a clathrin-mediated mechanism or via intermediary structures formed in a clathrin-independent manner—such as in bulk endocytosis, ultra-fast endocytosis, and formin-dependent pathways—synaptic vesicles fully collapse during fusion, leading to diffusion and intermixing of synaptic vesicle and plasma membrane lipids and proteins. The intermixing of synaptic vesicle and plasma membrane components not only poses a time constraint for synaptic vesicle biogenesis and reuse but also presents a cost-benefit dilemma. It would require a considerable amount of energy to retrieve all the necessary components in sufficient and adequate quantities and re-assemble a synaptic vesicle, especially since most synaptic vesicle proteins do not have classic sorting sequences (or other known motifs at present) and might require a combination of diverse adaptor proteins and other endocytic intermediates (like AP-2, AP-180, and iTRAPs). In fact, energy availability, in terms of mitochondria and ATP content, impacts the size and mobilization of the total pool and the readily releasable pool of synaptic vesicles ^25^. Taken together, for a synaptic vesicle that needs to respond and recover in the timescale of milliseconds, a full collapse mode of fusion with posterior recruiting and *de novo* assembly of components does not appear to be the most convenient mechanism.

The kiss-and-run pathway has been proposed as a mechanism that may preserve the molecular identity of synaptic vesicles. This pathway likely coexists with the previously discussed clathrin-independent modes of endocytosis but solves the time, energy, and identity issues associated with repeated synaptic vesicle biogenesis. Whereas clathrin-mediated endocytosis is an evolutionary preserved and ubiquitous mechanism with a widely described sequence of steps carried out by well-defined proteins with known morphological markers ^26^ and thus a variety of available molecular tools, kiss-and-run is a structurally distinct pathway that may involve different molecular partners, whose identities remain unknown, making it difficult to design tools and probes to examine it. Therefore, most of the existent and future experiments aimed to find evidence in support of a kiss-and-run type of fast retrieval and recycling of synaptic vesicles rely on electrophysiological and optical techniques improved to achieve very rapid time resolution.

## Kiss-and-run endocytosis

The kiss-and-run type of recycling preserves the shape and identity of synaptic vesicles, with no intermixing with plasma membrane or endosomal compartments. During this process, a fusion pore opens and closes transiently (that is, reversibly) without complete collapse of the vesicle, releasing neurotransmitter and regenerating a synaptic vesicle with no changes in molecular identity, which can be reused within seconds. The exocytosis process has been more easily observed in non-neuronal secretory cells because of better technical access to the fusion-retrieval coupling in these systems ^27– 29^. In these secretory cells, via direct visualization of morphological changes or trafficking of fluorescent probes along with electrophysiological capacitance measurements, it was possible to demonstrate the occurrence of transient exocytotic events consistent with the kiss-and-run model of fusion ^27– 29^. In small central synapses, however, the ability of capturing a fusion process that is considerably faster (about a few milliseconds) and smaller (about a couple of nanometers) compared with the non-neuronal counterparts has been technically difficult. Nevertheless, several studies have used exceedingly elaborate experimental settings to assess the existence and preponderance of kiss-and-run in neurons (see below). In addition to the lack of straightforward measurements of the kinetics and morphological characteristics of kiss-and-run, the actual molecular mechanism of this pathway remains to be determined. Accumulating evidence in recent years supports a clathrin-independent fast endocytic pathway that may either require or at least be regulated by the synaptic vesicle protein synaptobrevin, which is also a core component of the rapid Ca ^2+^-dependent fusion machinery ^30^. However, it is currently controversial whether kiss-and-run represents a reversible SNARE (soluble N-ethylmaleimide-sensitive factor attachment protein receptor)-mediated fusion event or almost instantaneous fission of vesicle-plasma membrane interface upon fully executed SNARE-mediated fusion (for a complete review of the role and mechanism of kiss-and-run, see 31).

The kiss-and-run type of endocytosis is not a new concept; it was proposed more than 40 years ago, shortly after the determination of the vesicular and quantal basis of neurotransmission, via pioneering experiments combining functional (electrophysiology) and structural (electron microscopy) analysis in the neuromuscular junction ^32, 33^. The peculiarity of this process was that synaptic vesicles maintained their curvature after fusion, arguing against full collapse. This allowed each vesicle to withstand several rounds of release, leading to quantal levels surpassing the total number of synaptic vesicles available per presynaptic terminal. Further experiments also showed that this type of recycling occurred in the same membranous region where fusion occurred—now called the active zone—and not at the periphery, as is the case for all of the clathrin-dependent and clathrin-independent pathways discussed in the previous section. Later on, the classification of synaptic vesicles as separate organelles and their exhaustive molecular characterization further supported the need of an endocytic mechanism that preserved or at least rapidly reconstituted their identity.

To summarize, the proposal of a kiss-and-run type of exo-endocytosis was quite attractive because it implicated many interesting attributes. First, it would save a considerable amount of energy to the neurons, avoiding the recruiting and retrieval of proteins from the plasma membrane and, partly, the refilling of synaptic vesicles. Second, it occurred in an extremely fast fashion, in the order of a few milliseconds, matching the rapid pace of synaptic transmission compared with other constitutive vesicle-trafficking events. Third, it would allow several rounds of exocytosis before depletion of neurotransmitter content, with high fidelity, avoiding overcrowding of the active zone.

Recent studies using fluorescently tagged synaptic vesicle proteins, however, challenged this notion and showed that at least the synaptic vesicle proteins synaptobrevin, synaptophysin-1, and synaptotagmin-1 can diffuse from the fusion site (while remaining confined to the nerve terminal) and endocytosis involves the recruitment of a distinct set of the same proteins previously resident on the plasma membrane of the periactive zone ^14, 34, 35^. These studies have two experimental aspects in common: they tested only synaptobrevin, synaptophysin-1, or synaptotagmin-1, and they applied relatively strong stimulations (that is, higher than 10 Hz). When tested at milder, single action potential stimulation paradigms, the same proteins revealed the coexistence of different endocytic mechanisms, with different kinetics as well as calcium dependencies ^36– 38^. A recent study expanded this concept by showing not only that slow and fast modes of endocytosis co-occur at different proportions depending on the level of neuronal activity but also that membrane itself (lipids) and proteins can be retrieved via different mechanisms ^39^. Furthermore, different synaptic vesicle proteins seem to be recycled through distinct mechanisms (particularly, synaptotagmin-1 and the vesicular glutamate transporter) ^9^, supporting the notion of parallel, independent endocytic pathways at the same presynaptic terminal. Nevertheless, a few synaptic vesicle proteins—namely synaptophysin-1, synaptotagmin-1, and the vesicular glutamate transporter—appear to dictate which of those modes of retrieval other proteins will undergo ^9, 38, 40^. Taken together, the dependence of the properties of these measurements on the identity of the tagged protein, along with the variabilities in stimulation paradigms, complicates a straightforward interpretation of the experiments in terms of synaptic vesicle recycling.

Since the early 1990s, the development of new optical probes like styryl dyes, quantum dots, and pH-sensitive green fluorescent protein (GFP)-tagged synaptic vesicle proteins allowed real-time *in vivo* measurement of fusion and retrieval kinetics, revealing that both processes are tightly coupled and occur in the timescale of seconds or less. Elegant experiments using quantum dots showed that a kiss-and-run type of endocytosis coexists with full collapse fusion in small central synapses, the proportion of both being dynamically regulated by the stimulus strength ^41^. Moreover, kiss-and-run occurs at mild stimulation intensities and preferentially involves the readily releasable pool of synaptic vesicles ^41, 42^. These findings were further supported by using quenching methods ^43^.

As mentioned before, classic clathrin-mediated endocytosis requires several seconds to even minutes to complete. For example, when the trafficking of a single cargo molecule is monitored by fluorescence, after fusion the residence on the plasma membrane and posterior recruiting and assembly of the clathrin machinery typically vary between 15 and 90 seconds (mean lifetime of 46 seconds ^44^). The rapid and high-fidelity retrieval of a complex organelle like a synaptic vesicle brings significant constraints to the classic clathrin-mediated endocytosis machinery, especially if *de novo* assembly of synaptic vesicles is involved in the process. However, the need of a rapid and reliable endocytic mechanism in neurons is not in itself proof of the existence of kiss-and-run; notably, several fast endocytic mechanisms have been proposed, or the clathrin pathway itself might be evolutionarily modified in neurons to perform faster and in synchrony with activity, as happens with the fusion machinery.

## Putative mechanisms underlying kiss-and-run

Although kiss-and-run is an attractive model with experimental substantiation in non-neuronal systems, morphological and functional characterization of this process in neurons has been technically challenging. In addition, the molecular mechanisms responsible for the triggering and regulation of this process are unknown. Since the fully assembled SNARE complex is highly energetically stable ^45^, it was proposed that kiss-and-run might rather be the result of an intermediary, reversible loose-SNARE complex (less stable) ^46^. However, whether these two forms of the SNARE fusion complex have the same or different molecular requirements (accessory and other SNARE-interacting proteins) is unknown. In non-neuronal secretory cells, for example, it was also proposed that the re-closure of the fusion pore is not the reversal reaction of the SNARE complex ^47^. After a synaptic vesicle has been docked and primed at the active zone, the fusion process starts, and if the vesicle undergoes a kiss-and-run type of exocytosis, there are mainly two ways to rapidly retrieve the synaptic vesicle: the mentioned reverse reaction of the SNARE complex or a different molecular machinery taking over the fast retrieval. The second proposal seems less likely since it will require longer times and there would be obvious steric constraints by requiring a large number of macromolecules around the fusion pore. Nevertheless, another molecular mechanism for fusion pore re-closure (equivalent to kiss-and-run) has been elegantly and meticulously demonstrated in chromaffin cells, where a hemi-fusion intermediary seems to be the critical step from which re-closure (fission) or full collapse proceeds in a Ca ^2+^- and dynamin-dependent manner ^22, 24^. The fusion of two membranes is an extremely conserved mechanism in living organisms and is mediated by two types of SNAREs: vesicle SNAREs, or v-SNAREs, which interact with their counterparts in the target membrane, or t-SNAREs. In neurons, the synaptic vesicle protein synaptobrevin-2/vesicle-associated membrane protein-2 associates with the plasma membrane proteins syntaxin-1 and SNAP-25 (25 kDa synaptosomal-associated protein). In this way, SNAREs form a complex that is extremely stable and resistant to detergents and proteases and that, thanks to the incorporation of the Ca ^2+^ sensor synaptotagmin-1, can respond rapidly and in synchrony to increases in Ca ^2+^ concentration by forcing the two opposed membranes into nanometer proximity, catalyzing an otherwise energetically unfavorable fusion ^45^. As mentioned before, this complex is extremely stable; it requires the function of an ATPase NSF (N-ethylmaleimide-sensitive factor) together with SNAPs (soluble NSF attachment proteins) for disassembly.

Owing to a lack of direct experimental proof, it is still difficult to envision how the SNARE-mediated fusion can be reversible. However, a considerable amount of work from different areas using various techniques points toward a reversible fusion process, where the fusion machinery (likely the SNARE complex or at least some of its components) would regulate both the “kiss” and the “run” ^27, 46^. For example, fusion experiments using nanodiscs composed of lipids and SNARE proteins showed that they are able to form pores that flicker (that is, open and close). These fusion pores are more stable, last longer, and have a different molecular architecture than random pores formed by lipid-only membranes ^48^. Amperometry studies in dopaminergic neurons also revealed the coexistence of at least two modes of release, and about 20% of them had flickering fusion pores which allowed for a longer and greater amount of neurotransmitter discharge ^49^. This reversible fusion pore is characterized by an all-or-nothing point; before that moment, the pore size can flicker (open and close several times), but once the all-or-nothing point has been reached (possibly a limiting pore size), the fusion proceeds irreversibly toward a full collapse with the plasma membrane ^27, 48^. The exact composition of the fusion pore is also a matter of debate but is believed to include a vast number of lipids and proteins which would allow not only opening and closing probability regulation but also geometry and net diameter determination ^50, 51^.

As discussed above, the switch between the full collapse and kiss-and-run fusion modes seems to be regulated by the release probability and the stimulation strength, indicating that there might a calcium-sensor protein involved in this switch. Additionally, the tension generated by the extent of vesicle attachment to the active zone matrix and the cytoskeleton has been proposed to impact the full collapse and kiss-and-run fusion ^31^, as has been demonstrated for dense-core vesicles ^23^. Molecularly, there is strong evidence that the transmembrane domains (TMDs) of SNARE proteins, specifically syntaxin and synaptobrevin, may play a central role in the formation (or nucleation) of the fusion pore ^48^. Even though SNAREs with their TMDs replaced by lipid chains still seem to be capable of catalyzing fusion (in a less efficient manner), the composition of the TMD (amino acid sequence, charge, and length) can regulate the fusion pore properties (size and stability) ^52– 54^. Particularly for very small fusion pores (~2 nm) like the ones established by synaptic vesicles, protein involvement seems to be crucial for pore stability. SNARE proteins have also been implicated in the regulation of fusion pore expansion kinetics (for a thorough review on fusion pores, see 55). Finally, kiss-and-run and SNARE complex disassembly may also involve the rapid action of the GTPase dynamin for fission of synaptic vesicles from the plasma membrane, which remains to be tested in neurons. In non-neuronal cells, dynamin-dependent as well as dynamin-independent kiss-and-run mechanisms have been proposed ^24, 47, 56^.

## The importance of synaptic vesicle molecular identity

Synaptic vesicles are divided into several pools with different propensities for fusion. Extensive work by several laboratories has demonstrated that this is not entirely related to their sub-synaptic localization but rather to variations in the vesicles’ molecular composition ^1, 57^. In addition, the fusion site in the active zone has been shown to differ between spontaneous and action potential-evoked forms of release ^58^, and activity regulates the localization and the number of fusion sites ^59^. Moreover, the recent discovery of the “trans-synaptic nanocolumns” demonstrated that presynaptic release sites are structurally aligned to postsynaptic receptors and associated signaling scaffolds, which can be dynamically regulated by activity ^58^. This spatial organization of the synapse can increase the potency of quantal release, segregate different modes of synaptic vesicle fusion, and enable their coupling to different signaling pathways, thus broadening the computational output of a single synapse. This concept has experimental support since spontaneous and evoked release has been shown to activate different populations of N-methyl-D-aspartic acid and α-amino-3-hydroxy-5-methyl-4-isoxazolepropionic acid receptors in multiple species ^60– 64^. Therefore, the preservation of molecular identity of synaptic vesicles after fusion can be critical in the maintenance of the size of the different vesicle pools and thus retain synaptic flexibility and enable signal multiplexing.

## Conclusions

Despite the consensus on the rapidity of neurotransmission response and recovery times, mechanisms underlying fast synaptic vesicle regeneration after fusion are still not fully understood. A number of studies have provided evidence for ultra-fast retrieval of fused synaptic vesicle membranes and proteins after fusion but have not yet provided a satisfactory solution to the problem of synaptic vesicle biogenesis. Since synaptic vesicles are highly specialized organelles with a unique protein composition, reassembling them is a daunting and energy-consuming task. The kiss-and-run hypothesis remains a viable option to account for their rapid reconstitution after fusion. Future studies that combine molecular specificity and fast timescale dynamic monitoring of synaptic vesicle trafficking are needed to answer this persistent fundamental question in synaptic physiology.

## Abbreviations

iTRAP, intrinsic trafficking partner; NSF, N-ethylmaleimide-sensitive factor; SNAP, soluble N-ethylmaleimide-sensitive factor attachment protein; SNARE, soluble N-ethylmaleimide-sensitive factor attachment protein receptor; TMD, transmembrane domain.
